# Ingestion of gelatinous candy, mimicking acute gastric bleeding

**DOI:** 10.1002/ccr3.2867

**Published:** 2020-05-27

**Authors:** Xavier Galloo, Maarten Vander Kuylen, Maurizio Tosi, Johan De Mey, Steven Raeymaeckers

**Affiliations:** ^1^ Department of Cardiology UZ Brussel Brussels Belgium; ^2^ Department of Surgery UZ Brussel Brussels Belgium; ^3^ Department of Anesthesiology UZ Brussel Brussels Belgium; ^4^ Department of Radiology UZ Brussel Brussels Belgium

**Keywords:** abdominal computed tomography, acute appendicitis, emergency radiology, gastric bleeding, gelatinous candy

## Abstract

A detailed medical history is vital in the correct interpretation of medical images: Peer‐to‐peer feedback and a thorough medical history can help avoid diagnostic pitfalls and unnecessary therapy.

## Graphical abstract

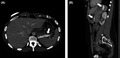

1

A 37‐year‐old woman presented with pain in the right iliac fossa. The clinician withheld the diagnosis of appendicitis. Contrast‐enhanced CT confirmed an engorged appendix with diffuse contrast‐enhancement, suggestive of acute appendicitis. Incidentally, however, the scan also revealed a homogeneous dense thick lining on the bottom of the stomach (Figure 1). The measured density of this layer fluctuated between 270 and 320 Hounsfield Units; values incidentally matching those of iodine contrast. No series of images had been acquired prior to the administration of IV‐contrast. The radiologist suggested an arterial bleeding of the gastric mucosa. The patient was re‐examined and then remembered having consumed some gelatinous candy in the waiting room, just prior to being scanned. A bag of said gummy bears was put in the scanner and indeed found to possess a comparable density to the collection found in her stomach (Figure 2). Quickly dissolved after ingestion, gelatine appears to settle as a thick layer on the bottom of the stomach.^1^ As no precontrast imaging was performed in light of radiation dose sparing, this then mimicked a stomach bleeding on the contrast‐enhanced images. Peer‐to‐peer feedback between radiologist and clinician as well as a full medical history spared this patient unnecessary therapy.

**Figure 1:**
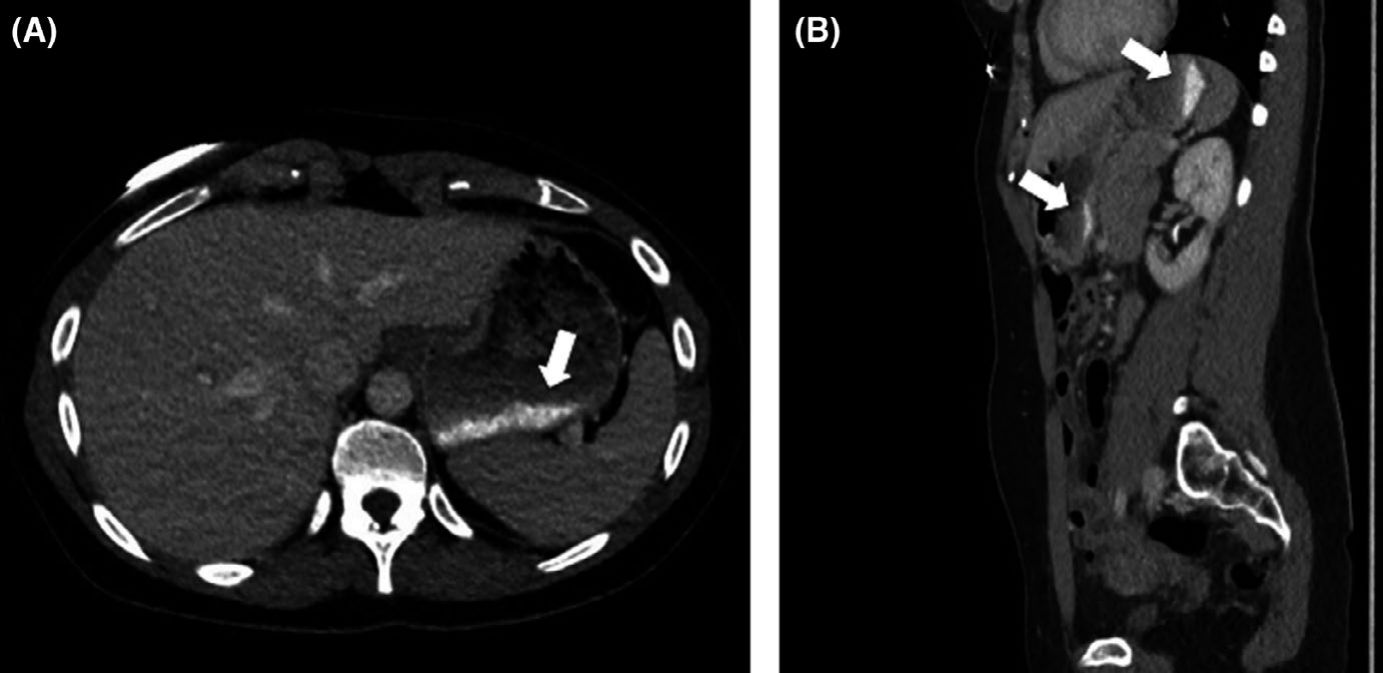
Contrast‐enhanced CT, axial (A) and sagittal (B) images. The bottom of the stomach is lined with an intensely dense content (270‐320 Hounsfield Units, white arrows), matching the density of iodine contrast

**Figure 2:**
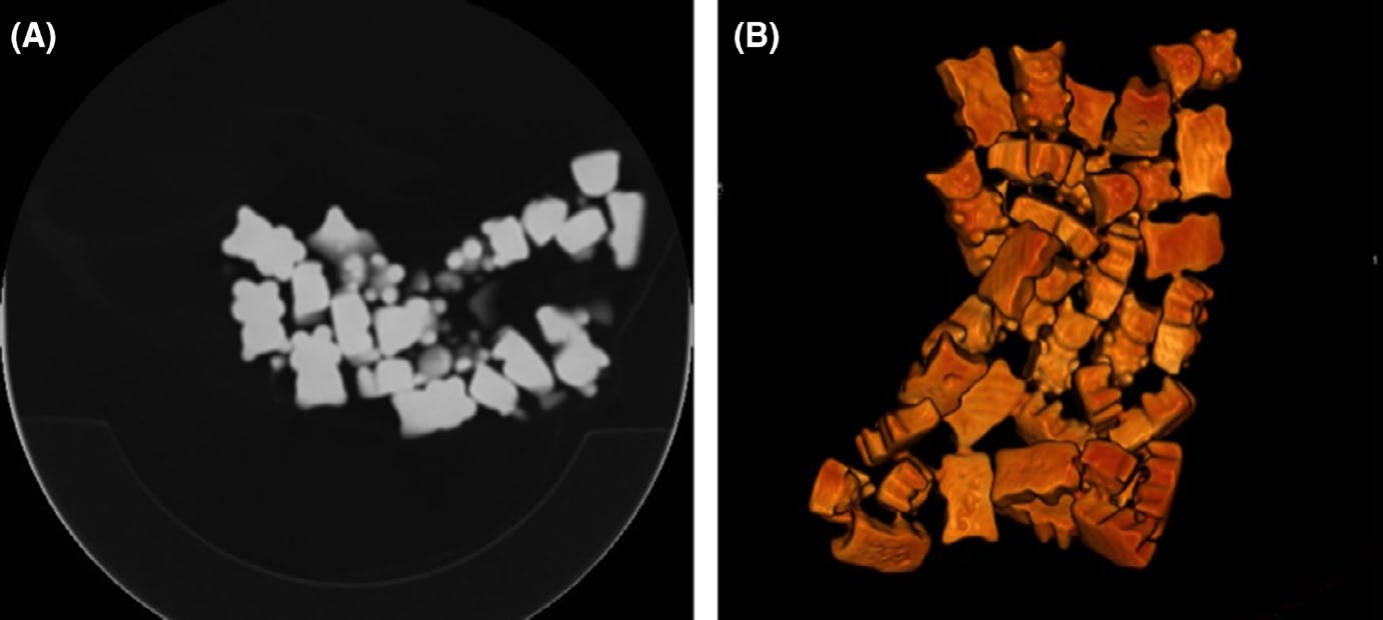
Noncontrast‐enhanced CT of a bag of gummy bear sweets, axial (A) and 3D reconstruction (B). Note the spontaneous dense aspect of the sweets

## CONFLICT OF INTEREST

None declared.

## AUTHOR CONTRIBUTIONS

XG: authored and revised the text. MVK: coauthored the text. MT: researched the literature. JDM: supervised and approved the article. SR: authored and revised the text, made the images, and supervised the article.

## ETHICAL APPROVAL

No approval warranted.

## INFORMED CONSENT

Informed consent was obtained from the patient included in the manuscript.
